# Incidence of acute kidney injury in COVID-19 infection: a systematic review and meta-analysis

**DOI:** 10.1186/s13054-020-03009-y

**Published:** 2020-06-16

**Authors:** Yih-Ting Chen, Shih-Chieh Shao, Cheng-Kai Hsu, I-Wen Wu, Ming-Jui Hung, Yung-Chang Chen

**Affiliations:** 1 Department of Nephrology, Chang Gung Memorial Hospital, Keelung, Taiwan; 2 School of Pharmacy, Institute of Clinical Pharmacy and Pharmaceutical Sciences, College of Medicine, National Cheng Kung University, Tainan, Taiwan; 3 Department of Pharmacy, Chang Gung Memorial Hospital, Keelung, Taiwan; 4 College of Medicine, Chang Gung University, Taoyuan, Taiwan; 5 Section of Cardiology, Department of Internal Medicine, Chang Gung Memorial Hospital, Keelung, Taiwan; 6 Kidney Research Center, Department of Nephrology, Chang Gung Memorial Hospital, Linkou, Taiwan; 7 Community Medicine Research Center, Chang Gung Memorial Hospital, Keelung, Taiwan; 8 Division of Nephrology, Department of Medicine, Chang Gung Memorial Hospital, No. 222, Maijin Rd., Anle Dist., Keelung, Taiwan

**Keywords:** Acute kidney injury, COVID-19, Incidence, Meta-analysis

Coronavirus disease 2019 (COVID-19), primarily affecting respiratory systems, has become pandemic and spread worldwide. Acute kidney injury (AKI) has been reported as a severe complication of COVID-19 with a higher risk of mortality [1], but the incidence of AKI among those infected with COVID-19 is currently only based on reports from small case series and retrospective studies [2, 3]. Therefore, in this work, we aim to perform a systematic review and meta-analysis of published articles to quantify the incidence of AKI in COVID-19 patients.

We performed a systematic search via PUBMED and EMBASE using the keywords “COVID-19” and “acute kidney injury” to identify relevant observational studies, such as case series and cohort studies published between 2019 and May 11, 2020. We also manually examined the reference lists of included studies and reviewed the AKI reports in epidemiological features and clinical courses of COVID-19 patients in high-profile general medicine journals (e.g., *BMJ*, *JAMA*, *Lancet*, and *NEJM*). Two independent reviewers (YTC and SCS) assessed articles, including title, abstract, and full text to determine whether studies were eligible for inclusion. In cases of divergences, results were discussed with a third reviewer (YCC). All statistical analyses were performed using MedCalc for Windows, version 15.0 (MedCalc Software, Ostend, Belgium). The incidence of AKI is expressed as proportion and 95% confidence interval (CI) using the random effects model and presented as a forest plot. We used the Cochran *Q* test to detect heterogeneity among studies, with a *p* value < 0.10 indicating significant heterogeneity. We calculated *I*^2^ statistic to measure the proportion of total variation in study estimates attributed to heterogeneity.

Of 65 articles screened, we excluded 45: 7 studies were duplicates, 8 studies were irrelevant, 9 studies failed to report the number of patients in the study cohort, and 21 studies did not report AKI data. Our final analysis included 20 articles comprising 6945 patients from China, Italy, the UK, and the USA. Demographic data for the included articles are summarized in Table 1. Notably, most of the studies (80%) were reported from China. We found the incidence of AKI was 8.9% (95% CI 4.6–14.5) in COVID-19 patients, but there was evidence of statistical heterogeneity among the studies with *I*^2^ = 97.8% and *p* < 0.001 (Fig. 1).

**Table 1:** Study characteristics

Author and year	City/country	Male (%)	Age (median)*	Settings	Patients with kidney transplantation (%)	Mechanical ventilation (%)	RRT (%)	ARDS (%)	Overall mortality (%)
Alberici 2020 [4]	Brescia/Italy	80	59	Hospitalization	100	10	5	55	25
Arentz 2020 [5]	Washington/USA	52	70	ICU	NR	71	NR	95	52
Banerjee 2020 [6]	London/UK	57	54	Hospitalization	100	29	43	29	14
Chen 2020 [7]	Wuhan/China	68	56	Hospitalization	NR	4	9	17	11
Chen 2020 [8]	Wuhan/China	62	62	Hospitalization	NR	6	1	72	41
Cheng 2020 [9]	Wuhan/China	52	63	Hospitalization	NR	14	NR	NR	16
Deng 2020 [10]	Wuhan/China	55	54	Hospitalization	NR	9	NR	48	48
Guan 2020 [11]	Wuhan/China	58	47	Hospitalization	NR	2	1	3	1
Guo 2020 [12]	Wuhan/China	49	59	Hospitalization	NR	24	NR	25	23
Huang 2020 [13]	Wuhan/China	73	49	Hospitalization	NR	10	7	29	15
Lei 2020 [14]	Wuhan/China	41	55	Hospitalization	NR	15	3	32	21
Richardson 2020 [15]	New York/USA	60	63	Hospitalization	NR	12	3	NR	21
Shi 2020 [16]	Wuhan/China	49	64	Hospitalization	NR	8	1	23	14
Wang 2020 [17]	Wuhan/China	58	54	Hospitalization	NR	NR	NR	10	6
Wang 2020 [18]	Wuhan/China	54	56	Hospitalization	NR	12	1	20	4
Wang 2020 [19]	Wuhan/China	53	51	Hospitalization	NR	19	NR	26	18
Yang 2020 [20]	Wuhan/China	67	60	ICU	NR	42	17	67	62
Zhang 2020 [21]	Wuhan/China	49	55	Hospitalization	NR	12	2	22	5
Zhang 2020 [22]	Zhejiang/China	51	45	Hospitalization	NR	1	0	2	NR
Zhou 2020 [23]	Wuhan/China	62	56	Hospitalization	NR	17	5	31	28

*In studies not reporting the median, age would be represented by the mean

*ARDS* acute respiratory distress syndrome, *ICU* intensive care unit, *NR* not reported, *RRT* renal replacement therapy

**Fig. 1:**
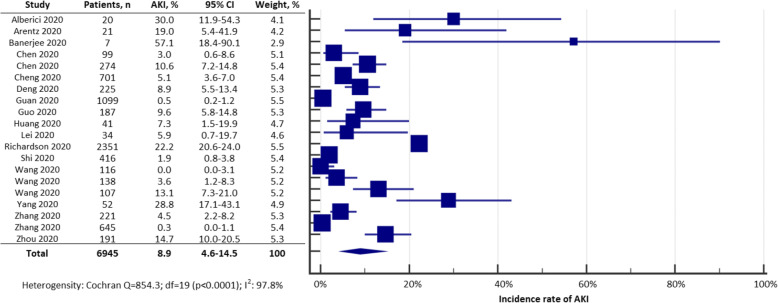
Forest plot of pooled incidence of AKI in COVID-19 patients from included studies

Previous studies reported the incidence of AKI largely from small case series or cohorts of COVID-19 patients, but our findings indicated that nearly 9 out of 100 developed AKI among a total of 6945 COVID-19 patients. This was close to the incidence rate of AKI in patients with community-acquired pneumonia [24].

Several mechanisms are possible for AKI in COVID-19 patients, including multi-organ dysfunction syndrome, SARS-CoV-2 direct kidney infection [25], AKI following acute respiratory distress syndrome (ARDS), infection-related generalized mitochondrial failure, and cytokine storm syndrome. Early recognition and treatment of AKI may limit associated complications such as long-term chronic kidney disease or end-stage kidney disease [26].

This study has several limitations. First, since the majority of included studies came from China and the USA, the generalizability of our findings into other countries may be limited. Second, clinical heterogeneity between studies should be noted, whereby detailed information on patient characteristics was lacking in the published articles. For example, two studies included patients post kidney transplantation, and the reported incidences of AKI were higher than in other studies which lacked information on how many patients had had kidney transplantation. With the disease burden of COVID-19 still increasing every day, we hope our synthesis can raise clinical awareness, early recognition, and intervention for AKI in patients hospitalized with COVID-19 for first-line healthcare providers.

## Data Availability

Not applicable.

## Abbreviations

AKI: Acute kidney injury
CI: Confidence interval
COVID-19: Coronavirus disease 2019
